# The Importance of Evaluating Digital Health in the Context of Addressing Climate Change

**DOI:** 10.1055/s-0046-1816596

**Published:** 2026-11-03

**Authors:** Mirela Prgomet, Farah Magrabi, Elske Ammenwerth, Michael Rigby, Kathrin Cresswell, Stephanie Medlock, Elisavet Andrikopoulou, Andrew Georgiou

**Affiliations:** 1 Australian Institute of Health Innovation, Macquarie UniversitySydneyAustralia; 2 UMIT TIROL, Private University for Health Sciences and Health TechnologyHall in TirolAustria; 3 Keele University, School of Social, Political and Global Studies and School of Primary, Community and Social CareKeeleUnited Kingdom; 4 The University of Edinburgh, Usher InstituteEdinburghUnited Kingdom; 5 Amsterdam UMC location University of Amsterdam, Department of Medical InformaticsMeibergdreef 9AmsterdamNetherlands; 6 School of Computing, University of PortsmouthPortsmouthUnited Kingdom

**Keywords:** Digital Health, Climate Change, Evaluation Studies, Health Policy

## Abstract

**Objectives:**

As climate change increasingly threatens societal wellbeing, including public health and healthcare systems, the environmental impact and role of digital health technologies as mitigating agents warrants critical exploration. This paper examines evaluation of the utility of digital health to address climate-related healthcare challenges and discusses the environmental sustainability of digital health.

**Method:**

vA perspective paper based on a narrative review of literature alongside expert perspectives from the International Medical Informatics Association (IMIA) Working Group on Technology Assessment and Quality Development in Health Informatics and the European Federation for Medical Informatics (EFMI) Working Group for Assessment of Health Information Systems.

**Results:**

Digital health technologies hold immense potential to identify climate-related health challenges through predictive analytics, reduce the environmental impact of the healthcare sector through virtual care delivery, and equip healthcare systems to better respond to and navigate disaster scenarios. However, the increasing use of technologies raises concerns about their environmental impact due to high energy consumption and e-waste generation. It is, therefore, essential that their development, implementation and optimization is evaluated with a focus on environmental sustainability.

**Conclusion:**

To ensure the sustainable integration of digital health technologies, a holistic evaluation approach is necessary. Informaticians, healthcare providers, technology developers, and policymakers must collaborate to establish standardized frameworks that incorporate sustainability metrics across the entire lifespan of digital health initiatives. By doing so, digital health can have a positive impact on healthcare challenges while minimizing its environmental footprint.

## 1. Introduction

Climate change represents one of the most significant global challenges of the 21st century, with profound implications for human health [ 1 ]. Rising temperatures, changing weather patterns, and an increase in the frequency of extreme events, such as heatwaves, hurricanes, wildfires, and floods, are exacerbating health issues worldwide [ 2 – 4 ]. These health issues include heightened incidences of heat-related illnesses, such as heat exhaustion and heatstroke [ 5 , 6 ], respiratory conditions due to worsening air quality from air pollutants and wildfires [ 7 ], and the proliferation of infectious diseases, such as vector-borne disease like malaria and dengue as altered precipitation patterns create favorable conditions for vectors to thrive and expand into new regions [ 8 , 9 ]. Climate change, particularly the rise in extreme weather events, exacerbates life-threatening droughts, crop failures, and livestock mortality, thereby heightening water and food insecurity [ 2 , 10 ]. This situation often necessitates population displacement, which in turn adversely impacts the public health of the receiving communities. Vulnerable populations, including the elderly, children, those with chronic health conditions, and individuals in low-income communities, face the brunt of climate change challenges, further straining already burdened healthcare systems [ 2 , 3 ]. The need to bolster the resilience of healthcare systems by means of tracking, monitoring, and responding to climate-related issues is, therefore, paramount and it is here that digital health can play a critical role [ 11 ].

Digital health encompasses a broad spectrum of information and communication technologies, including telemedicine, mobile health applications, electronic health records (EHRs), wearable devices, and artificial intelligence (AI)-based tools, all of which aim to improve care delivery and patient outcomes. These technologies hold immense potential to address the multifaceted health challenges posed by climate change by enabling predictive analytics, enhancing virtual accessibility to care, and streamlining disaster response [ 12 ]. However, currently, the healthcare sector is responsible for approximately 4.6% of global greenhouse gas emissions with digital health technologies contributing to these emissions [ 2 ]. The increasing adoption of digital health technologies, and their application in addressing health challenges posed by climate change, necessitates balancing the environmental impacts of digitization to ensure these technologies alleviate, rather than exacerbate, healthcare’s carbon footprint [ 13 ]. Thorough assessment of digital solutions, to determine their capacity to mitigate climate-related health impacts while minimizing their own environmental footprint, is therefore essential to ensure their environmental sustainability [ 14 ]. This should ideally occur at the outset of a technology initiative and requires dual evaluation – assessing the utility of digital health in addressing climate change issues, as well as assessing their carbon footprint – to underpin the sustainable and enduring integration of digital health into healthcare systems.

In this paper, we aim to draw attention to the importance and key considerations for evaluating digital health in the context of climate change. We first examine evaluation of the utility of digital health technologies to address climate change (Section 2). Within this section we draw on three approaches to leveraging digital health to address climate change: predictive analytics, virtual accessibility, and disaster response [ 12 ]. We give an overview of the application of these digital health solutions and discuss metrics to evaluate their effectiveness and value as tools to address climate-related healthcare challenges. In the subsequent section (Section 3), we discuss the environmental footprint of digital health and highlight frameworks to underpin evaluation of digital health sustainability.

## 2. Evaluating Utility of Digital Health to Address Climate Change

There are many ways that digital health can be leveraged to advance the healthcare system’s preparedness for the potential impacts of climate change. Below, we describe examples from three key areas of application – predictive health data analytics, virtual accessibility to care, and disaster response – and metrics to evaluate their utility in tackling climate-related healthcare challenges. A summary is provided in Box 1.

**Box 1:** Summary of example frameworks and metrics to evaluate the utility of three approaches to leveraging digital health to address climate-related healthcare challenges

Area of Application	Predictive Health Data Analytics	Virtual Accessibility to Care	Disaster Response
Examples of Digital Health Technology	Artificial intelligence, machine learning	Telehealth, wearable devices	EHRs, patient portals, mobile devices
Example Framework to Assess Utility	AI in Healthcare Framework [ 15 ]	Virtual Care Evaluation Framework [ 16 ]	Digital Health Evaluation Framework [ 17 ]
Examples of Additional Climate-Related Metrics for Evaluation	Reliability in predicting and managing climate-related health risks and outcomes Facilitation of timely interventions Identification of targeted intervention through early detection Reduction of healthcare system burden Integration of data sources for comprehensive and actionable insights Computational resources and energy consumption	Reduction in travel Decreases in operational demands of healthcare facilities Potential risks like missed diagnoses leading to subsequent in-person visits or admissions Equitable access (climate vulnerable populations) and accessibility in resource-constrained settings Adaptability to diverse cultural and linguistic contexts	Reliability under stress/challenging conditions Scalability across diverse geographic and demographic contexts Readiness for rapid deployment Ability to maintain continuity of care and support data-driven decision-making during disasters Capacity to modify strategies, scale up interventions, and allocate resources in response to changing conditions

## 2.1. Predictive Health Data Analytics

One of the critical applications of digital health technologies in the context of climate change is health data analytics. Health systems amass vast quantities of data for monitoring, improving and informing policy and care delivery across healthcare settings [ 18 ]. Advanced tools, such as AI and machine learning, can be used to harness these large datasets to model and predict health challenges triggered by climate change [ 19 , 20 ]. For instance, AI/machine learning algorithms have been used to: identify patterns in disease outbreaks, such as the correlation between rising temperatures and increased malaria transmission in tropical regions [ 21 ]; forecast vector numbers, aiding in early warning systems for arboviral disease outbreak risk [ 22 ]; identify eco-climatic drivers of virus outbreaks, enabling timely alerts for surveillance and control measures [ 23 ]; and predict the risk of adverse health outcomes from climate-responsive extreme weather events, including heatwaves and floods [ 24 ]. Similarly, models can monitor the impact of climate-related health impacts on vulnerable populations enabling targeted interventions, such as monitoring respiratory health issues from dust storms in Africa [ 25 ].

The capabilities of AI/machine learning make them incredibly valuable digital health tools to tackle climate-related health issues. Nevertheless, the increasing use of these advanced analytics tools in healthcare also prompts significant concerns regarding their environmental impact given the substantial computational resources and energy consumption they require in order to process immense volumes of data [ 26 ]. Both impact and process evaluation is therefore crucial to ensure that AI/machine learning tools identify and signpost potential effective action to deliver sustainable and significant improvements to public policies and healthcare, thereby balancing their environmental impact. To date, evaluations have largely focused on the accuracy of data analytics model predictions [ 15 , 27 ]. While evaluating accuracy is fundamental, to determine the impact of AI/machine learning tools in healthcare settings and their utility in addressing climate-related challenges, a thorough evaluation that goes beyond computational performance is necessary [ 28 ]. Factors that could be considered include the extent to which AI/machine learning can: reliably predict and manage climate-related health risks and outcomes; enable early preventative care and targeted intervention through early detection; facilitate timely interventions and reduce the burden on healthcare systems; and integrate data from various sources to provide comprehensive and actionable insights. Such factors assessing climate-related issues need to be linked to process evaluations, underpinned by frameworks such as the AI in healthcare framework outlined by Park *et al.* [ 15 ] or the theory-informed health information technology evaluation frameworks synthesized by Cresswell *et al.* [ 29 ], to assess how effects are achieved and monitor potential adverse consequences. Undertaking comprehensive evaluations, underpinned by frameworks and involving multidisciplinary evaluators (e.g., computer science, medical informatics, social science, environmental science), would verify that data analytic technologies are not only scientifically robust and achieve their aim of predicting climate-related health effects, but also improve their integration into healthcare settings and ensure their sustainability and scalability.

## 2.2. Virtual Accessibility to Care

Virtual care, including telehealth/telemedicine and remote patient monitoring, has emerged as a transformative solution for reducing the environmental impacts of healthcare. By facilitating care monitoring via digital health tools (such as wearable devices) and care access via remote consultations, virtual care reduces the need for patient travel and decreases the operational demands of healthcare facilities [ 12 , 30 , 31 ]. This shift is particularly valuable in mitigating carbon emissions from transportation, which constitutes a significant portion of the healthcare sector’s environmental impact [ 32 ]. A systematic review of 18 articles found that virtual care consultations avoided an annual carbon footprint ranging from 0.7 to 4.35 metric tonnes of CO2 emissions [ 33 ]. Virtual care can also facilitate care accessibility during extreme weather conditions when travel or medical evacuations are not possible [ 34 ] or emergency healthcare services are overburdened [ 35 ].

While there is strong evidence that virtual consultations significantly reduce carbon emissions by minimizing travel for in-person appointments, current research often overlooks system factors related to implementing virtual healthcare. In their systematic review of the role of virtual care in developing environmentally sustainable healthcare, Pickard Strange *et al.* found limited consideration of broader factors such as patient suitability, clinical indications, and organizational infrastructure that influence the adoption and effectiveness of virtual care [ 31 ]. Additionally, the carbon footprint of the entire clinical pathway, including potential risks like missed diagnoses leading to subsequent in-person visits or admissions, was identified as not being thoroughly examined [ 31 ]. Climate change also disproportionately affects vulnerable populations [ 3 ], including those in low-income regions and marginalized communities. Virtual care must be evaluated for its ability to serve these populations equitably. This includes examining accessibility in resource-constrained settings and adaptability to diverse cultural and linguistic contexts. Thus, for comprehensive evaluation of the effectiveness, safety, and sustainability of virtual care, it is essential to consider a broad scope of perspectives including implementation, process, outcome and environmental impacts. For implementation, formative (process) evaluations can guide optimal design and delivery of virtual care, assessment of stakeholder needs and issues surrounding equity, literacy and bias [ 36 ]. Metrics for evaluating effectiveness and quality of virtual care include safety outcomes, timeliness and accessibility of care, engagement, healthcare usage, user satisfaction, and barriers and enablers [ 37 ]. To quantify environmental impact, the life cycle assessment (LCA) method can be used to measure digital health emissions [ 38 ]. LCA considers the environmental impacts of products, processes, or organizational activities throughout their entire life cycle [ 39 ]. Thus, LCA goes beyond the energy consumed for travel and encompasses energy savings from digitizing work processes, building operations, and the energy consumed by digital health tools [ 12 ]. Integration of outcome and LCA evaluations with formative evaluations can subsequently provide a holistic view of the utility of virtual care as a tool to address climate change challenges. The Virtual Care Evaluation Framework [ 16 ], developed by Alberta Health Services, also provides a comprehensive guide designed to evaluate virtual care programs and services and uses a structured approach to assess the effectiveness, safety, and sustainability of virtual care. The framework emphasizes considering unintended consequences and using a logic model to guide the evaluation process from the outset to ensure effective and sustainable integration of virtual care into healthcare settings [ 16 ].

## 2.3. Disaster Response – Adaptation and Resilience Planning

Adaptability is a crucial characteristic of a resilient healthcare system. The capacity to modify strategies, scale up interventions, and allocate resources in response to changing conditions is vital for effectively tackling emerging challenges [ 40 ]. In this context, digital health technologies could play a pivotal role in supporting adaptation strategies to climate change and enhancing the resilience of healthcare systems [ 41 ]. Disasters, particularly those exacerbated by climate change, create complex and dynamic information environments. Data flows originate from a myriad of diverse organizations, including healthcare providers, emergency services, and government agencies. Digital health tools, such as electronic health records (EHRs), patient portals, telemedicine, point of care testing devices, and mobile health applications, may serve as crucial assets in managing this data and delivering critical information to decision-makers [ 12 , 42 ]. During climate-related emergencies, such as hurricanes, floods, or wildfires, digital health facilitates continuity of care through access to patient records [ 43 ]. For instance, portable EHRs can allow displaced individuals to maintain consistent medical attention even when traditional healthcare infrastructure is compromised [ 44 ].

Currently, there is limited evidence evaluating the utility of digital health technologies for climate-related disaster management. Evaluation of digital health for disaster response, adaptation, and resilience planning requires a framework-based approach that encompasses structural factors (such as infrastructure), patient factors (such as locality), and medical system factors (such as capacity and capability) [ 45 ]. The Digital Health Evaluation Framework, an amalgamation of existing evaluation frameworks, aims to facilitate evaluation of digital health at a regional level [ 17 ]. Key dimensions to consider include reliability under stress, scalability across diverse geographic and demographic contexts, and readiness for rapid deployment. Stress-testing digital health systems allows assessments of functionality under the challenging conditions often present during disasters, such as power outages, network disruptions, and surges in data traffic [ 41 , 46 ]. As disasters often affect diverse populations across wide geographic areas, digital health tools should be assessed for scalability to ensure they meet the needs of varying population sizes and adaptability to meet different cultural and linguistic contexts [ 47 , 48 ]. The rapid deployment of digital health technologies is critical during disasters, but this requires integration of data sources across a range of settings at scale including healthcare and public services. Evaluations should assess the processes of integration (both technically and sociotechnically), pre-deployment planning (including the establishment of interoperability standards and emergency response protocols), and how well systems can streamline activation during crises [ 49 ]. Digital health systems should also be evaluated based on their ability to maintain continuity of care and support data-driven decision-making during disasters [ 44 ]. This involves assessing their capability, accuracy, timeliness, and the usability and reliability of the information they provide. For example, geospatial information systems (GIS) integrated with health data can help identify areas most in need of medical intervention, guiding resource allocation [ 50 ]. Undertaking evaluation of adaptability and resilience of digital health for disaster management is critically needed to ensure these technologies can uphold care standards and inform effective responses, to equip healthcare systems to better navigate the complexities of disaster scenarios.

## 3. Evaluating the Environmental Impact of Digital Health

Healthcare is a major source of greenhouse gas emissions [ 2 ]. Every aspect of healthcare contributes to carbon emissions, from the energy used in healthcare facilities to the transportation of patients and staff, as well as the various products and services that support healthcare operations [ 51 ]. Digital health is no exception. In this section, we discuss the environmental footprint of digital health and strategies proposed in literature aimed at reducing this impact. We then focus on highlighting frameworks for evaluating digital health sustainability.

### 3.1. Environmental Footprint of Digital Health

Digital health technologies undoubtedly play a pivotal role in addressing current and future climate change challenges in healthcare. The environmental footprint of digital health arises mainly from the energy consumption of data centers and networks supporting applications like EHRs, patient portals, AI-based tools, telemedicine, mobile health applications, and wearable devices contributing to greenhouse gas emissions through electricity use, device manufacturing, and disposal [ 51 – 53 ]. It is, therefore, vital that the healthcare and informatics communities espouse climate consciousness otherwise digital health risks contributing to, rather than mitigating, healthcare’s carbon footprint [ 13 , 54 ]. For example, as discussed above, AI and machine learning for predictive analytics can be valuable for identifying disease outbreak patterns, forecasting vector numbers, predicting adverse health outcomes from extreme weather events, and monitoring climate-related health impacts on vulnerable populations [ 21 – 25 ]. However, the infrastructure – data centers, servers, and communication networks – powering advanced technologies, such as AI/machine learning, consume vast amounts of energy contributing to a substantial carbon footprint [ 26 ]. As these digital health technologies grow more complex and data-intensive, the energy demands are anticipated to further rise. Using the LCA methodology, Luccioni *et al.* determined the carbon emissions of training a 176 billion parameter large language model to be 50.5 tonnes when considering all processes, from equipment manufacturing to energy consumption during operations [ 55 ]. While this carbon footprint may appear relatively modest, the widespread adoption of AI/machine learning models in future healthcare could lead to significant cumulative environmental impacts. Solutions such as reducing computational demand by developing energy-efficient models through techniques like model compression; adopting green computing practices with energy-efficient hardware and optimized software; transitioning data centers to renewable energy sources; and conducting LCA to evaluate and minimize the environmental footprint throughout the lifespan of the technology can help mitigate these impacts [ 26 ]. It is, therefore, essential to carefully consider the need and use of models in each situation to ensure they provide sensible, significant, and sustainable improvements to the quality and safety of care delivery. The use of simpler models was demonstrated to significantly reduce carbon emissions without compromising performance in a modelling study of the environmental sustainability of machine learning in digital pathology [ 52 ]. The study also projected future emissions and emphasized the critical role of renewable energy, energy-efficient hardware, and less computationally intensive models in mitigating future emissions.

The rapid pace of technological innovation also exacerbates the issue of electronic waste (e-waste) as outdated devices, from smartphones to diagnostic equipment, are discarded [ 30 ]. E-waste is one of the fastest-growing waste streams in the world [ 56 ]. Aside from the physical waste they create, electronic devices often contain toxic materials, which can pose significant risks to the environment and human health [ 56 ]. Proper e-waste management practices, such as robust recycling programs and repurposing outdated devices, are essential to mitigate this issue [ 26 ]. Furthermore, many computing devices are replaced before the end of their useful lifespan [ 13 ]. Extending the usage period of these devices can reduce the materials and energy needed for manufacturing devices, while the procurement of more resilient devices, that can be updated rather than discarded, could decrease the amount of e-waste generated. The i-CLIMATE framework, developed to aid the reduction of environmental pollution from healthcare, advocates for the establishment of a circular economy for health technology and integrating the principles of reusing, refurbishing, or repurposing technology [ 13 ]. For example, by upgrading components instead of replacing hardware, healthcare organizations can reduce e-waste and emissions. Additionally, at procurement stage, healthcare organizations should aim to contract certified “green” vendors offering recycling programs and prioritize Energy Star ratings to maximize energy efficiency [ 13 , 26 ]. Undertaking LCA of devices can facilitate decisions about procurement, usage, maintenance, and disposal. This involves assessing the environmental impact of equipment production, energy consumption during use, and proper disposal methods to reduce the carbon footprint of digital health technologies.

### 3.2. Frameworks for Evaluating Environmental Sustainability of Digital Health

Evaluating the environmental sustainability of digital health initiatives is crucial to ensure that the advancement of health technology aligns with environmental goals. This evaluation is complex and requires consideration of several factors throughout the entire lifecycle of the digital health initiative, from production to disposal [ 57 , 58 ]. In recent years, a number of frameworks and approaches have emerged that can guide sustainability evaluations.

Examples include:

- Life cycle assessment (LCA) framework [ 59 ] – provides a means to evaluate the carbon footprint of digital health by assessing the environmental impact of manufacturing, utilizing, and disposing of digital health tools;
- Maturity model for sustainable healthcare [ 60 ] – facilitates the identification of the environmental footprint of a healthcare setting by examining governance, organization structures (including tools and technology), processes, and outcomes and control;
- Green IT frameworks (e.g. Green-Mission framework [ 61 ], Green IT Maturity framework [ 62 ]) – these frameworks focus on assessing and identifying sustainable interventions to minimize environmental impact;
- Framework for energy efficiency testing to improve environmental goal of software (FEETINGS) [ 63 ] – guides energy consumption measurements for software to evaluate energy efficiency;
- i-CLIMATE framework [ 13 ] – while not an evaluation framework, it provides an actionable structure to facilitate reduction of environmental pollution from healthcare, which can inform evaluation metrics to assess healthcare sustainability practices.

Key evaluation considerations across these frameworks includes assessment of energy efficiency and e-waste management. Incorporating sustainability metrics into the design, implementation, and scaling of digital health systems is essential. However, as highlighted by Lokmic-Tomkins *et al.* [ 58 ] in their review of existing environmental assessment frameworks, methods, and tools for evaluating the carbon footprint of digital health interventions, there is currently a lack of standardized methods or validated tools to systematically assess the environmental sustainability of a digital health intervention throughout its entire lifecycle. This highlights a critical area of research that demands urgent attention. Existing frameworks provide a robust foundation from which elements can be drawn and integrated to develop a more comprehensive and inclusive framework that encompasses the assessment of environmental sustainability of digital health technologies from production to disposal. Undertaking this work should be a multidisciplinary effort involving researchers, technology developers, healthcare professionals, environmental scientists, and policymakers to ensure the development of a framework that is scientifically rigorous, practically applicable and guides the healthcare sector towards more sustainable practices. By adopting such an evaluation framework at the outset of an initiative, digital health can be positioned not only as a tool for improving healthcare delivery but also as a critical component in healthcare’s effort toward environmental sustainability.

## 4. Recommendations and Conclusions

The environmental consequences of climate change, from climatic variability to extreme weather events, can all impact human health. Healthcare must strive to minimize its environmental footprint and address the health impacts of climate change through innovative, sustainable, and scalable solutions. Digital health technologies present a unique opportunity to enhance the resilience of healthcare systems against climate change while advancing environmental sustainability goals. They hold immense potential to identify climate-related health challenges through predictive analytics, reduce the environmental impact of the healthcare sector through more environmentally considered care delivery including virtual care, and equip healthcare systems to better respond to and navigate the complexities of disaster scenarios. But their integration must be evaluated with a dual focus on effectiveness and environmental sustainability.

Based on our above overview of literature, we put forward four recommendations on integrating evaluation into every stage of digital health initiatives:

First, prior to implementing any digital health technology, healthcare organizations should adopt sustainability metrics into their digital health procurement processes and conduct thorough evaluation to understand both the potential utility and environmental impacts of the technology. This ensures that technology not only meets healthcare needs but also aligns with sustainability goals from the outset.

Second, implementation of digital health technologies should be evaluated to determine their real-world capacity to mitigate climate-related health impacts while minimizing their own environmental footprint. Regular monitoring should be undertaken to track progress and identify any emerging issues. This allows for timely optimizations, ensuring that the technologies remain effective and environmentally sustainable over time.

Third, multidisciplinary stakeholders should be involved in evaluation processes, including informaticians, healthcare professionals, environmental experts, patients, and policymakers. This diverse participation ensures that multiple perspectives are considered, including technical, clinical, social, ethical, and environmental aspects, leading to a more comprehensive understanding of the technology’s impact.

Fourth, policymakers and researchers should prioritize the development of standardized frameworks for evaluating both the utility of digital health technologies in addressing climate-related health challenges and their environmental impact across their entire lifecycle. These frameworks should include criteria for assessing the effectiveness of digital tools in mitigating health issues exacerbated by climate change and quantifiable metrics to measure the environmental sustainability of digital health initiatives. 

By adopting these recommendations, healthcare systems can systematically evaluate and enhance the environmental sustainability of digital health initiatives, ensuring that they contribute positively to both health outcomes and environmental goals.
